# Coatings for Cardiovascular Stents—An Up-to-Date Review

**DOI:** 10.3390/ijms25021078

**Published:** 2024-01-16

**Authors:** Alexandru Scafa Udriște, Alexandra Cristina Burdușel, Adelina-Gabriela Niculescu, Marius Rădulescu, Alexandru Mihai Grumezescu

**Affiliations:** 1 Department 4 Cardio-Thoracic Pathology, “Carol Davila” University of Medicine and Pharmacy, 050474 Bucharest, Romania; alexandru.scafa@umfcd.ro; 2 Department of Science and Engineering of Oxide Materials and Nanomaterials, Politehnica University of Bucharest, 011061 Bucharest, Romania; alexandra.burdusel@upb.ro (A.C.B.); adelina.niculescu@upb.ro (A.-G.N.); agrumezescu@upb.ro (A.M.G.); 3 Research Institute of the University of Bucharest—ICUB, University of Bucharest, 050657 Bucharest, Romania; 4 Department of Inorganic Chemistry, Physical Chemistry and Electrochemistry, University Politehnica of Bucharest, 1-7 Polizu St., 011061 Bucharest, Romania

**Keywords:** cardiovascular stents, stent coatings, polymers, titanium oxide, magnesium alloys

## Abstract

Cardiovascular diseases (CVDs) increasingly burden health systems and patients worldwide, necessitating the improved awareness of current treatment possibilities and the development of more efficient therapeutic strategies. When plaque deposits narrow the arteries, the standard of care implies the insertion of a stent at the lesion site. The most promising development in cardiovascular stents has been the release of medications from these stents. However, the use of drug-eluting stents (DESs) is still challenged by in-stent restenosis occurrence. DESs’ long-term clinical success depends on several parameters, including the degradability of the polymers, drug release profiles, stent platforms, coating polymers, and the metals and their alloys that are employed as metal frames in the stents. Thus, it is critical to investigate new approaches to optimize the most suitable DESs to solve problems with the inflammatory response, delayed endothelialization, and sub-acute stent thrombosis. As certain advancements have been reported in the literature, this review aims to present the latest updates in the coatings field for cardiovascular stents. Specifically, there are described various organic (e.g., synthetic and natural polymer-based coatings, stents coated directly with drugs, and coatings containing endothelial cells) and inorganic (e.g., metallic and nonmetallic materials) stent coating options, aiming to create an updated framework that would serve as an inception point for future research.

## 1. Introduction

Cardiovascular diseases (CVDs) are becoming more prevalent, being the leading cause of premature death and disability in humans worldwide. CVDs greatly increase healthcare costs and significantly impact the general population from a socioeconomic point of view [1,2,3,4,5,6]. The well-acknowledged etiological risk factors, that, when combined, account for almost 90% of the risks of CVDs, include hypertension, hyperlipidemia, diabetes mellitus, obesity, smoking, poor diet, and physical inactivity [2,7,8,9,10]. The main approaches for preventing and treating CVDs assume the administration of lipid-lowering medications, antihypertensives, and antiplatelet and anticoagulant treatments. Moreover, positive lifestyle modifications are recommended. Notwithstanding the efficacy of these strategies, there are still significant gaps in managing CVDs [2,11].

Using fluoroscopic and/or intravascular guidance, special devices known as stents can be put into the damaged channel to restore normal blood flow and prevent the additional serious effects of vascular constriction. The tiny, intricate, hollow structures with cylindrical shape arranged into a sequential ring structure with several struts and connecting components allow stents to keep the human artery’s passage through the body open [6,12,13]. Thus, cardiovascular stents are life-saving technologies that can reduce long-term mortality and morbidity and should be ranked among the top ten medical innovations of the twenty-first century [2,14,15].

Initially, simple balloon angioplasty was utilized to treat abrupt and threatening artery closure, and bare-metal stents were the backup intervention option. On the other hand, the documented favorable results prompted the standard of treatment for percutaneous coronary intervention (PCI) to be changed to stent implantation. The use of the initial generation of these devices was often limited by in-stent restenosis, which led to the failure of the current stent or a reintervention using a different stent, regardless of the history of clinical safety and efficacy [16,17,18]. Due to the shortcomings of bare metal stents (BMSs), new stent materials, designs, surface treatments, and coatings have been investigated to enhance the device’s functionality and, in particular, to reduce the rate of in-stent restenosis. Drug-eluting stents (DESs) have marked a significant advancement in stent development. Years after their design, DESs were introduced as a remedy for these problems and are now an essential treatment choice for people with coronary artery disease. Coronary DESs have significantly decreased vascular stent restenosis and decreased the need for additional revascularizations, which has revolutionized PCI therapy and has led to them being approved as the current standard [6,18,19,20,21].

An essential component of DESs is the stent coating that incorporates the medication. The coating must ensure structural integrity, consistent dosing, and controlled release kinetics. As the interface between the stent and the vascular tissue, the material used to coat these biomedical devices must be biocompatible, non-thrombogenic, non-inflammatory, and non-toxic to cells while promoting arterial healing and re-endothelialization [22,23,24,25,26]. Considering these requirements, one extensively studied possibility is to use polymeric coatings to aid in loading the drug onto the stent. These coatings prevent the drug from being washed off during device deployment, provide precise loading and controlled drug delivery, and, ideally, enhance the stent’s biocompatibility after the drug has been washed out [27]. Other coatings of an organic nature have also been rendered promising for optimizing stent performance. Materials like heparin, hyaluronic acid, fibrin, and endothelial cells have also been reported in the literature as useful for fabricating stent coatings [28,29]. Great promise has also been envisaged by tackling inorganic materials: metallic (e.g., titanium-based, magnesium-based, and layered double hydroxide stent coatings) and non-metallic (e.g., phosphate-based, fluoride-based, and graphene oxide coatings) [28,30].

In this context, this paper presents the advancements in cardiovascular stents, briefly overviewing their evolution over the years and further detailing organic and inorganic coating possibilities. Specifically, various stent coating materials are described, ranging from synthetic and natural polymers to metallic and nonmetallic inorganic alternatives for stent optimization. In this respect, relevant English language papers retrieved from scientific databases, such as Science Direct, PubMed, and Google Scholar, have been reviewed, offering a comprehensive outlook of the topic.

## 2. Cardiovascular Stent Evolution

The first technology ever invented for the treatment of coronary artery disease was balloon angioplasty, which was first performed in 1977. Between the introduction of this intervention and 1990, 40 to 60 percent of restenosis cases were related to the initial angioplasties, which were based on balloon expansion in the artery and had problems with elastic rebound and neointimal hyperplasia using only a balloon to illustrate the occurrence of restenosis after angioplasty [6,31].

It is noteworthy that there are second-generation balloons designed to treat in-stent restenosis that are coated with medications, typically paclitaxel. In this situation, the drug must be transferred quickly during the balloon’s brief contact with the vessel wall, which lasts about a minute. This technique has attracted the attention of some researchers because it reduces the risk of bleeding, prevents the risky presence of a foreign object in the body, and minimizes side effects. However, this method was never fully developed for several reasons, most notably the promising outcomes of stents [32].

**Figure 1:**
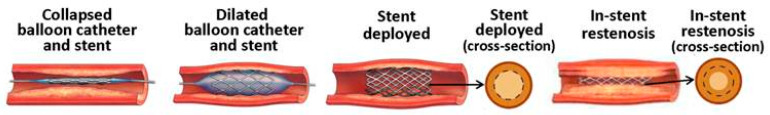
In-stent coronary restenosis after coronary angioplasty with bare metal stents. Reprinted from an open-access source [34].

Additionally, due to the restenosis of the treated artery following balloon deflection, BMSs were introduced in the 1990s to address this flaw in ballooning and line the artery wall. Between 1991 and 2003, this reduction in restenosis to 20–30% was made possible by eliminating elastic recoil. But BMSs had a disadvantage: it was a foreign object to the immune system, which could respond to this intrusive object in various ways. For example, white blood cells known as macrophages could accumulate around the stent, and nearby smooth muscle cells (SMCs) could proliferate and obstruct the endothelialization process. The luminal region narrows after vascular SMCs move from the media to the intima, multiply, and form an additional layer of cellular matrix there (intimal hyperplasia). Likely, the leukocytes that stick to the activated endothelium and prevent it from healing cause thickened intima [33]. These undesirable processes eventually lead to in-stent restenosis (shown in Figure 1).

Because of these harmful side effects, incorporating the stents into the body was made possible by combining the medications. After that, a new generation of stents known as DESs in 2003 revolutionized how restenosis in the artery was treated. To treat the stenting area, this generation carries restorative medications with them. These stents are covered in a polymer layer with an active ingredient that lowers neointimal hyperplasia [35]. The incidence of restenosis decreased to about 3–20%. The issues of denuded intima and the associated inflammation and thrombosis remain unresolved despite DESs having primarily dealt with the problem of restenosis [19].

According to a report from 2010, stent thrombosis occurs after 0.3–0.6% of DES stenting procedures each year, followed by an increase in human mortality of 10–30%. The main causes of inflammation and late thrombosis are polymer-induced inflammation, stent malapposition, and late or incomplete re-endothelialization [32].

**Figure 2:**
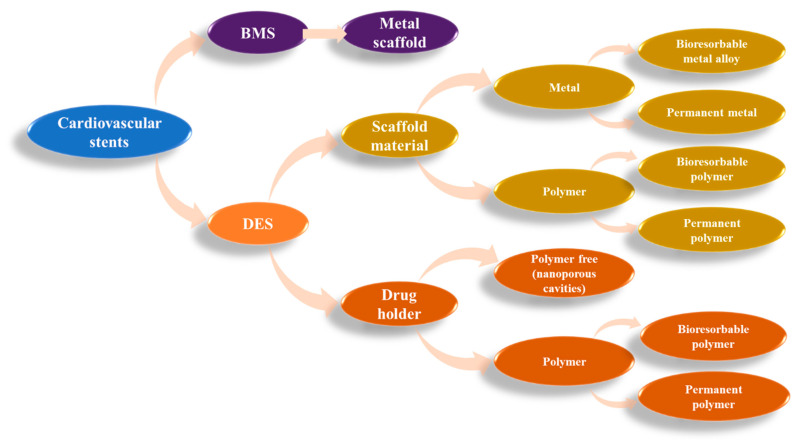
Schematic of the material choice in the various stents. Created based on information from [34,37,38].

Scientific progress is being made to address this issue. These procedures, which date back around 40 years, are still being refined today: balloon angioplasty, bare stent, drug-eluting stent, and bioresorbable stent. The many stents are compiled from the material perspective in Figure 2 [36].

## 3. Organic Coatings for Stents

### 3.1. Synthetic Polymer-Based Coatings

Polymers are large molecular compounds with repeating structural units, typically connected by covalent chemical bonds. Polymers are used extensively in cardiology, particularly coronary vascular intervention, as coating matrices for drug-eluting stents and stent platforms (scaffolds). Synthetic polymers such as poly(ethylene-co-vinyl acetate) (PEVA), poly(n-butyl methacrylate) (PBMA), or tri-block copolymer poly(styrene-b-isobutylene-b-styrene) (SIBS) were utilized in the Cypher and Taxus Express stents, which were the first generation of DES stents. However, it was believed that these polymers were to blame for the persistent hypersensitivity of the artery wall, which might have postponed the stent struts’ endothelizalization process and raised the risk of stent thrombosis [39]. More biocompatible polymers with a lower propensity for inflammatory response and delayed vascular healing were created to get around these restrictions. The polymer used in both the PROMUS and Xience everolimus-eluting stents consists of two layers: a fluorinated polymer called poly(vinylidene fluoride-co-hexafluoropropylene) (PVDF-HFP) covers a base layer of PBMA [40]. Because albumins stick to the surface of fluoropolymers, they reduce prothrombotic proteins, including fibrinogen, and platelet adhesion and activation [41]. A hydrophobic C10 polymer, a hydrophilic polyvinylpyrrolidone polymer, and a hydrophilic and hydrophobic polyvinylpyrrolidone C19 polymer make up the multi-component BioLinx polymer used in the Resolute zotarolimus-eluting stent. Hydrophilic proteins generate a hydrophilic layer on hydrophobic BioLinx components, resulting in the amphiphilic nature of the entire polymer surface and perhaps reducing the adhesive capacity of other plasma proteins [42].

A local inflammatory response could be triggered by the permanent presence of even biocompatible polymers. Bioresorbable polymers were created to boost biocompatibility and speed up the endothelization process. After the intended amount of time, the bioresorbable polymer dissolves, leaving behind a metallic scaffold comparable to BMS and lessening the possibility of polymer interactions with artery tissue. Additionally, some polymers are only used on the abluminal side of the stent, which lessens the possibility of plasma and polymer molecules interacting. This is thought to further reduce the risk of potential adverse events by minimizing the polymer burden and lowering drug concentrations, making them, in theory, more biocompatible than a conventional DES. However, extensive clinical trials have not yet demonstrated this [43].

Additionally, essential to enhancing device biocompatibility, is stent coating. There are many coating methods, but the most well-liked ones are direct-write inkjet, electrospinning, dip coating, and spray coating. Additionally, a variety of applied polymer types, such as polymer-free stent platforms (PF), biodegradable polymers (BPs), and durable polymers (DPs), are used today. By removing the polymer’s constant exposure to vessel healing, which occurs in DP-DES stents, biodegradable polymers were created to reduce inflammatory reactions and lower the instances of late and very late stent thrombosis. However, recent studies comparing the clinical outcomes of BP-DES and DP-DES produced mixed results, and some of these studies could not prove that BP-DES was superior [44].

Theoretically, with both BP-DES and PF-DES stents, the incidence of adverse events associated with polymers should decline or disappear entirely. The primary building blocks of BP-DES polymers include poly(L-lactic acid), poly(D,L-lactide), polycaprolactone (PCL), and poly(lactide-co-glycolide) or poly(D,L-lactide-co-caprolactone (PLCL)] [45]. The last polymer breaks down into carbon dioxide and water, and the first three into lactic acid. Comparing BP-DES stents to DP-DES stents, the ability to cause inflammation is even lower. Such polymers are frequently thin (for example, PLLA is 7.5 microns thick at the abluminal stent surface and 3.5 microns thick at the luminal surface in Orsiro SES), applied only to the abluminal surface, and have a quicker drug-eluting time, which speeds up the endothelialization of the struts. It is still unclear whether BPs are associated with better outcomes compared to DP stents. The frequency of major adverse cardiac and cerebrovascular events (MACCE) or clinically driven target vessel revascularization (CD-TLR) was lower for BP-DES stents compared to DP-DES stents after 5 years, even though several studies have not demonstrated the superiority of BP-DES over DP-DES stents [46].

In addition to permanent polymers, biodegradable polymers are a main topic of current research. Since they break down once their purpose is served, using them may help prevent negative outcomes like in-stent restenosis, late stent thrombosis, and hypersensitivity reactions [47].

DESs are specialized vascular stents that enable controlled local drug delivery to reduce or prevent in-stent restenosis due to increased SMC proliferation. Additionally, second- and third-generation DESs currently use biomimetic polymers like phosphorylcholine (PC), poly(vinylidene fluoride)-hexafluoropropylene (PVDF-HFP), or the BioLinx polymer because they do not obstruct stent reendothelialization. In addition, biodegradable polymers, such as PLGA and poly(lactic acid) (PLA), have undergone extensive research to enhance their biocompatibility and properties [48]. DESs are predicted to result in lower stent thrombosis because the polymeric coatings eventually deteriorate and become BMSs. The next generation of DESs with an even greater impact on endothelialization and arterial healing will result from intensive work on stent development. The dip- and/or spray-coating techniques are used in current DES polymer coating technology [49]. These techniques work well for coating stents with strongly lipophilic medications like sirolimus but not with water-soluble medications or DNA. It has been reported that plasmid DNA or oligonucleotide-coated stents have been made with a water-soluble polymer. These stents demonstrated poor therapeutic outcomes and delivery efficacy for clinical use [50].

Nanotechnology is anticipated to significantly impact the development of effective and secure drug delivery systems. A polymeric nanoparticle (NP) made of the biodegradable polymer PLGA can entrap hydrophilic agents (protein, oligonucleotide, DNA, and the like), penetrate cellular membranes via endocytosis, and deliver the therapeutic agents it has encapsulated into the cellular cytoplasm was previously described. The body slowly hydrolyzes the PLGA, metabolizes it, and excretes it. The benefits of the PLGA NPs include the effective intracellular delivery of various therapeutic agent classes and the ability for prolonged intracytoplasmic release [50,51,52]. No existing technology has been able to coat metallic stents with an active coating of NPs up until this point. Currently, dip coating is used to develop polymer carrier coatings on the surfaces of vascular stents; however, this method has shortcomings such as non-uniformity, blockage, and winding. Electrostatic spray deposition reduces the polymer solution into minuscule droplets that stick to the substrate surface when exposed to a high electric field, resulting in uniform and smooth coatings. This technique is widely used in the chemical industry, machinery, electronic data, medical data, micromedical devices, and other fields. In a recent study, PLGA was added to a DES as a coating, and it was discovered that it had a powerful inhibitory effect on the growth of granulation tissue. In some recent research, the degradation rates of the coating were found to be lower than the matrix metal by electrophoretically depositing a gelatin GNS/CTS (gelatin nanospheres/chitosans) composite coating on a WE43 substrate. It was found that in patients with true coronary bifurcation lesions, rapamycin drug-coated double stents more efficiently controlled the levels of serum adiponectin, GDF-15, and Cys-C. The drug coating had a higher biocompatibility and a lower cytotoxicity. Due to its special characteristics compared to durable polymers in drug delivery, biodegradable PLGA coating for applications in drug-eluting stents has attracted growing interest. This is done to reduce stent-related side effects. The study conducted by Jia et al. [53] validated a mathematical model to describe how PLGA stent coating erodes and degrades, along with the coupled drug release. For PLGA mass loss, an analytical expression was developed, making several experimental studies in the literature predictable. The change in the number-average degree of polymerization (or molecular weight) is also the subject of an analytical model. The drug transport model takes into account simultaneous drug diffusion through the solid polymer and the liquid-filled pores in the coating, and it derives an efficient drug diffusivity model by taking into account variables like the change in polymer MW, the change in stent coating porosity, and the drug’s partitioning between the solid and aqueous phases [53].

The results of the randomized controlled trials completed so far are inconsistent or insufficient to answer the question of how effective and safe they are. However, several studies show that, in terms of mortality, stent thrombosis, and long-term efficacy, patients treated with polymer-free stents exhibit clinical outcomes comparable to those treated with durable polymer DESs. On the other hand, it has been claimed that new-generation DESs have been shown to be superior or noninferior to durable DESs in terms of the safety and efficacy [34].

In addition to the coatings above, essential in improving biocompatibility, polymers have another responsibility: transporting and releasing therapeutic agents locally to the injured area of arteries [36]. DESs’ first and second generations are currently coated with nonbiodegradable polymers on the US market to regulate the drug-eluting profile. In the first generation of DESs, the stent coating of the Cypher^®^, a sirolimus-eluting stent (Cordis, Bridgewater, NJ, USA), is a polymer blend of poly(ethylene-co-vinyl acetate) and poly(n-butylmethacrylate) loaded with sirolimus. In contrast, the coating of the Taxus^®^, a paclitaxel-eluting stent (Boston Scientific, Marlborough, MA, USA), is poly(styrene-b-isobutylene-b-styrene) loaded with paclitaxel. According to the clinical results, both DESs and BMSs can significantly reduce restenosis. However, patients 18 months to 3 years after the implantation of Cypher and Taxus reported an increase in the rate of MI and mortality. In the second generation of DES, stent coatings of the Xience V^®^, an everolimus-eluting stent (Abbott Vascular, Temecula, CA, USA), and Endeavor^®^, a zotarolimus-eluting stent (Medtronic Vascular, Milpitas, CA, USA), are fluoropolymer and phosphorylcholine, respectively. Compared with BMSs, target vessel revascularization (TVR) was dramatically reduced by both the Endeavor and Xience V stents. It was reported in the SPIRIT III study that Xience V stents could reduce angiographic late loss without an increase in ST, compared with Taxus stents, and also showed superiority in the COMPARE trial, with a lower rate of MIs and TVR [28].

Despite claims that some nonbiodegradable polymer-coated DESs are safe in the long term, there is still concern about the inflammatory response. As a result, biodegradable polymers are being investigated and considered for use in drug delivery and storage. PLA, PGA, and their copolymer, PLGA, which can be completely degraded and metabolized by the body, are currently the most widely used polymers. Numerous biodegradable polymer-coated stents are currently undergoing clinical trials [54].

Although NPs have been widely used in numerous drug delivery systems, few studies have examined the effects of coating stent surfaces with NPs. Recently, using cationic electrodeposition coating technology, an active coating of NPs was successfully applied to the surfaces of metallic stents, and the in vivo viability of this NP-eluting stent was characterized and assessed [55]. The researchers concluded that the NP-eluting stent, compared to the dip-coated polymer-eluting stent, is a potentially innovative platform with unique characteristics in vascular compatibility and an effective drug delivery system. A further intriguing NP-mediated drug delivery system consists of 304-grade stainless steel and magnetic NPs loaded with endothelial cells (ECs). After injection, the PLA-modified magnetic NPs loaded with ECs were targeted by a magnetic field gradient towards the stent surface rather than being coated directly on the stent surface. This allows for repeated dosing and artificial endothelialization [56]. Despite the reported positive outcomes, further research into this approach in animal studies and clinical trials is still needed because the concept is still in its experimental stages [45].

A safer alternative for stent implantation is bioresorbable vascular scaffolds (BVSs), which the body can fully absorb without the need for follow-up procedures to remove permanent stents. This lowers the patient’s risk of developing additional chronic diseases. Biodegradable stents integrate bioresorbable and bioabsorbable stent technologies to eradicate the risk of late stent thrombosis. In contrast to bioabsorbable stents, which only absorb the drug and leave a bare metal scaffold behind, bioresorbable stents fully absorb all of their constituent parts—drugs and/or scaffolds—within the human body. Only one BVS, developed by Abbott et al. using poly (lactic acid) (PLLA) as the stent platform, has received FDA approval. Positive vessel remodeling and plaque regression have been reported for this BVS during the resorption process, which occurs one to five years after implantation. However, compared to metallic stents, polymeric stents generally have a lower tensile strength, reduced stiffness, and reduced ductility. Additionally, there have been reports of late thrombosis clinical problems with polymeric DESs. Reports have also indicated that stent thrombosis may be brought on by PLGA scaffolds. Studies have shown that the components of bioresorbable scaffolds can cause hypersensitivity reactions and localized foreign body reactions. Several proteins, including fibrinogen, fibronectin, and albumin, adsorb on the surface of the stent after it is implanted. Because of the protein adsorption on the implant’s surface, macrophages identify it as a foreign substance. When implanted scaffolds are present, macrophages differentiate and become giant cells in response to the foreign body. Thirteen acidic breakdown products (such as lactic and glycolic acid) produced during the PLGA degradation of polymer scaffolds lower the pH of the surrounding tissues, which can cause in vivo foreign body reactions and inflammation. PLGA degradation products following implantation have raised biocompatibility concerns, rendering this polymer material no longer biologically inert. However, since most cases of hypersensitivity or allergic reactions have not been reported, metallic biomaterials are highly popular in biomedical applications (for example, magnesium) [57,58,59].

### 3.2. Natural Polymer-Based Coatings

#### 3.2.1. Chitosan-Based Coatings

Chitosan (CS), a type of naturally occurring polysaccharide polymer that has good biodegradability and can encourage cell adhesion and proliferation, is made up of glucosamine and N-acetylglucosamine units linked by one to four glycosidic bonds. Additionally, CS exhibits a strong antibacterial property due to the numerous positive charges that cover its surface [60,61,62]. To investigate the impact of CS coatings on magnesium alloy corrosion, CS coatings were deposited on two different magnesium alloys, AM 20 and WE 43, using various pre-treatment techniques, including un-treatment, acid treatment, coupling agent treatment, and glutaraldehyde treatment. According to that study, the WE 43 alloy, which contains rare earths, is more suitable as a biomaterial. Additionally, the CS coating slowed the corrosion rate, but the results varied depending on the type of treatment used. To achieve Mg alloys’ controllable degradation, a more thorough investigation of the in situ degradation mechanism is essential. However, the limited application of CS coatings is due to the weak interfacial adhesion force between them and the bare substrate. These use the electrostatic attraction between CS and poly-l-glutamic acid (PGA) to create a functionalized coating with corrosion-resistant and antimicrobial properties on the magnesium alloy AZ31. The obtained (CS/PGA) demonstrated good corrosion resistance when submerged in SBF solution compared to the Mg alloy substrate because of the pH-buffering action of the weak–weak polyelectrolyte pair [63]. The Mg alloy was also given good antibacterial properties, which work through the contact-killing method. To investigate corrosion resistance and biocompatibility, a recent study used the LbL technology to synthesize a bioactive chitosan-functionalized graphene oxide/heparin (GOCS/Hep) multilayer on the AZ31B Mg alloy treated with a combination of a surface chemical treatment with an in situ self-assembly of 16-phosphoryl-hexadecanoic acid [64]. The study found that the Mg alloy had excellent in vitro corrosion resistance thanks to the GOCS/Hep composite coating. The GOCS/Hep coating, which created excellent blood compatibility, significantly reduced platelet adhesion and activation. The GOCS/Hep coating also supported the expression of nitric oxide (NO) and vascular endothelial growth factor (VEGF) on the surface of the attached ECs, which in turn improved EC adhesion and proliferation [65].

#### 3.2.2. Heparin-Based Coatings

Heparin is a drug and a naturally occurring glycosaminoglycan that is also referred to as unfractionated heparin (UFH). Heparins are classified as anticoagulants because they rely on the activity of antithrombin. It is specifically used to treat unstable angina and heart attacks. It can be administered intravenously or through a skin injection. Its anticoagulant properties are also used in kidney dialysis machines and blood specimen test tubes [66]. Bleeding, pain at the injection site, and low blood platelets are typical side effects. Thrombocytopenia brought on by heparin is one of the serious side effects. Those with impaired kidney function require more attention [67].

Heparin has been liberally used on the surfaces of vascular implants due to its anticoagulant properties and inhibitory effect on arterial smooth muscle cell proliferation. The heparin coating of stents, in particular, is efficient and secure [68]. However, other randomized clinical human trials have not found differences between heparin-coated and uncoated stents regarding the rate of thrombosis and restenosis [69]. For instance, in a trial using heparin-coated and uncoated Jostent^®^ (Jomed International AB, Helsingborg, Sweden), stent thrombosis and restenosis were 1.9 versus 1.3%, and 33.1 versus 30.3%, respectively. Furthermore, no effect on the angiographic or clinical events was noted when comparing heparin-coated stents with BMSs for treating stenoses in small coronary arteries [67].

When mixed with aspirin plus ticlopidine or clopidogrel, heparin-coated stents may solve subacute stent thrombosis and offer stronger antiaggregation in patients at high risk for thrombotic complications. The effectiveness of stents coated with heparin is still up for debate, though [70].

A prospective, randomized trial was designed in a previous study to compare the potential clinical and angiographic benefits of stenting (i.e., heparin-coated stenting vs. standard balloon angioplasty). Haude et al.’s study aimed to treat stenoses in small native coronary arteries. The goal of the heparin coating was to reduce thrombosis in stents, which was thought to happen more frequently in small vessels [71].

A study by Christensen and colleagues [72] showed a very low 0.5% rate of thrombotic events following both elective bare and heparin-coated stenting; however, treatment with ticlopidine or clopidogrel was started in the majority of cases right away following stenting, and glycoprotein IIb/IIIa blockers were not routinely given. These thrombotic event rates range from 0.8% to 1.9% and are comparable to those of other heparin-coated stent trials. Although the Corline heparin coating used in the current trial differs from other heparin coatings in terms of the conditioning layer, the heparin attachment, and the antithrombin III-binding activity, the additional antithrombotic benefit of any heparin coating is debatable, given the low thrombotic event rate following the elective bare stenting of small vessels with the adjunctive administration of aspirin and ticlopidine or clopidogrel. This message cannot be generalized to other situations, such as stenting for acute coronary syndromes, which includes myocardial infarction with an elevation [72].

#### 3.2.3. Hyaluronic Acid-Based Coatings

Hyaluronic acid (HA) is a glycosaminoglycan widely distributed throughout the body’s tissues and primarily found in the extracellular matrix. It is not sulfated. In a baboon thrombosis model, HA-coated stainless steel stents and tubes have been shown to significantly reduce the formation of platelet thrombus [73]. Furthermore, HA cross-linking with N-(3-dimethylaminopropyl)-N′-ethyl carbodiimide-coated stents had a favorable inherent antiproliferative effect regarding neointimal formation after local vessel wall injury (overstretch model) and resulted in a decreased inflammatory response in undiseased pig coronary arteries. However, evaluating airway stents coated with a cross-linked HA derivative in a rabbit airway model of subglottic stenosis in a later animal study produced different outcomes. According to the data, this stent coated with an HA derivative may help lessen stenosis in patients who do not have airway injuries, but it does not significantly improve healing in posttraumatic models [74].

#### 3.2.4. Fibrin-Based Coatings

Fibrin is a fibrous protein that is created when thrombin cleaves fibrinopeptides from fibrinogen during blood clotting [75]. In a porcine coronary injury model, the effectiveness and safety of a fibrin-film-covered stent have been assessed. When added to metal stents, fibrin functions as an excellent biocompatible and biodegradable polymeric coating. Such a “hybrid stent” offers particular benefit to the arterial injury site by covering and delivering drugs to the entire lesion surface. This is because the fibrin film provides complete endoluminal paving with anti-thrombogenic or antiproliferative therapy delivery. The efficacy and safety of a fibrin-coated stent need to be further assessed in human clinical trials despite the results of animal studies suggesting fibrin is a promising stent coating [76].

Three pairs of polypeptide chains connected by 29 disulfide bonds make up the large, intricate, fibrous glycoprotein known as fibrin. Hemostasis and angiogenesis depend on the deposition of fibrin. In particular, fibrin increases the production of extracellular matrix proteins by cells, creating a basement membrane necessary to develop stable microvascular networks [77]. Thus, transient fibrin deposition stimulates cell adhesion, spreading, migration, and alignment. Earlier discoveries point to increased endothelialization following iron stent corrosion. Therefore, it was concluded that the fibrin deposition on the surface of the corroded iron stent was caused by rapid endothelialization. This hypothesis was tested using an innovative and convincing arterial implantation model. Clinical observations showed that when the stent was placed at the artery bifurcation, the endothelialization process was significantly slowed down. Endothelialization was hindered by the abrupt change in the blood flow direction at the artery bifurcation and the associated shear stress, which was much higher on the vessel wall in this position. The researchers selected the iliac artery bifurcation and inserted a corroded stent to address the effects of fibrin deposition on endothelialization. Additionally, the S316L stent and the iron stent with fibrin coating were employed as controls. Surprisingly, during the initial experimental period, the fibrin-coated stents showed rapid endothelialization. More fibrin deposits were found on the surface of the corroded iron stent with a longer implantation time. Neointimal coverage of the corroded stent was subsequently started. On the other hand, the S316L stent showed much less fibrin deposition and had a smooth surface with minimal endothelial cell coverage. In short, it was demonstrated that iron stent corrosion promoted fibrin deposition, which raised the endothelialization rate by introducing the bifurcation artery implantation model [77].

The basis for endothelial cell adhesion and growth is provided by the extracellular matrix protein fibrin. Polytetrafluoroethylene prosthetics with fibrin coating promoted endothelialization. Additionally, under blood flow conditions, fibrin coating aids in the maturation and stabilization of endothelial cells. In fact, several blood proteins, including fibronectin, von Willebrand factor, albumin, and VEGF, can bind to fibrin. Fibrin plays a role in inflammation, thrombosis, and cell growth. Following fibrin deposition on the corroded stent surface, endothelialization was encouraged. As a result, it was suggested in a recent study that an attachment of fibrin improves endothelialization by increasing endothelial cell adhesion. By introducing fibrin-coated stents, new ex vivo experiments were carried out to test this hypothesis. Furthermore, a parallel-plate flow chamber system was employed, and the co-culture medium was exposed to dynamic flow conditions to replicate the actual conditions of blood flow. On the fibrin-coated stent surfaces, endothelial cell adhesion was faster. Furthermore, the stent was found to cause significant alterations in mRNA levels related to cell adhesion. Quantitative measurements were made for cell proliferation in addition to endothelial cell adhesion. However, no discernible differences were seen in the early stages of the experiment. Contrarily, the number of cells in the fibrin-coated plate started to rise with extended incubation, and there were roughly 200% more endothelial cells in the fibrin-coated plate than in the control plate. Based on these findings, it was shown that fibrin deposition increased endothelialization by encouraging the adhesion and proliferation of endothelial cells [78].

Fibrin has been discovered to have many promising medical uses, including promoting operative hemostasis during cardiovascular surgery and improving the endothelialization of peripheral vascular grafts in vitro. Native fibrin is deposited at the site of arterial injury and, in part, determines the neointimal response, according to studies in the porcine coronary model. This evidence served as the impetus for the initial investigation into how fibrin-coated stents affected the arterial wall [79]. Twenty animals received 34 of these stents using the same porcine coronary model, and there were no immediate complications. After 28 days, a follow-up angiography verified the patency of all 31 stented coronary segments still in place. The fibrin was fully endothelialized and retained its structural integrity in every instance. Importantly, the fibrin stent did not cause an excessive neointimal response or a foreign body reaction, and the local vascular integrity was kept. Initial reports of using autologous vein grafts to achieve full natural stent coverage are encouraging. Twenty-seven autologous vein graft stents were implanted in the iliac arteries of the porcine model, with the uncoated stents serving as control stents. There were seven days to six months of follow-up. After two months, all the autologous vein graft stents remained patent and integrated into the arterial wall. Additionally, no heightened neointimal response was noticed. Seven patients have had these stents implanted by the same group without any problems during a mean follow-up of up to four months [80].

According to the study of McKenna et al., when compared to a BMS, the fibrin stent alone does not lessen the neointimal response to coronary injury. However, fibrin is the best candidate for local drug delivery because it can completely and safely cover the stented coronary segment and degrade gradually for one to three months. The ability to deliver site-specific therapy is constrained by BMSs only covering less than one third of the lesion surface area upon deployment. However, complete arterial coverage makes avoiding major coronary side branches practical [80].

The way fibrin is formulated affects how quickly it is reabsorbed. Therefore, altering the formulation should make it possible to regulate the biodegradation rate. The matrix size can also be changed, and microcapsules that deliver drugs locally can be added. This method would increase the fibrin stent’s ability to suppress the neointimal response—which was still noted with the standalone device. Because of worries about viral transmission, the Food and Drug Administration outlawed commercially available fibrin products in 1978. Before use, the fibrin film used in the current stent is dehydrated and gamma-sterilized, eliminating any risk of infection. The human donors who provided the fibrinogen are also screened for viral infections [81].

### 3.3. Polymer-Free Stents

In PF-DESs, the drug is directly bonded to the stent’s scaffold. With the aid of metal scaffolds or simply coating the surface (typically the abluminal part), modern technology enables the creation of porous nanostructures on stents, which can then be coated with materials such as drugs. To elute a drug in a controlled manner, various structures and surfaces (such as nanotubes, nanoleaves, nanograss, nanoflakes, nanopillars, and nanowires, among others) can be fabricated on metallic surfaces. A particular titania nanoleaf structure among the different surface types has demonstrated higher cytocompatibility and hemocompatibility in vitro, with high endothelialization and low smooth muscle cell proliferation [82,83].

Different surface characteristics, such as topography, chemistry, or roughness, are characterized by different influences on plasma, proteins, and cells. This information may be crucial in upcoming studies. A drug can be placed on a nanotexture’s porous surface, and hydrophilic/hydrophobic nanostructures can control the kinetics of drug release. The elution profile of an antiproliferative drug with a shorter elution time—for example, 100% of a drug is eluted in 1 month in BioFreedom^®^ (Singapore)—is a crucial component of PF stents. Platelet cells fail to adhere to the micro/nanostructure, according to research. Stent surfaces that have undergone technological modification can mimic the extracellular matrix and thus aid in controlling vascular cell adhesion and proliferation. Without the polymer, stents do not interact with living tissue, which minimizes the risk of inflammation, postponed endothelial healing, and restenosis. Recent research has not yet demonstrated the superiority of PF-DES over other stent types. Large studies have revealed that PF-DESs are more likely to cause MACCE, TLR, and in-stent late lumen loss than BP-DESs. Furthermore, two significant meta-analysis studies showed no difference between DP-DESs and PF-DESs in terms of MI, cardiac death, all-cause death, stent thrombosis, TLR, TVR, and diameter stenosis. The efficacy and safety of manufactured stents, including PF-DESs, will be improved in coronary stents as part of future development [32,84].

A typical illustration of a microporous stent that elutes sirolimus is Yukon^TM^ (Translumina, Hechingen, Germany). The 316L stainless steel stent’s surface is mechanically altered to create the micropore, which serves as a drug reservoir and eliminates the need for a polymer. The microporous stent surface has an average roughness of 1.96 ± 0.21 μm, as measured by a perthometer. Utilizing specialized equipment, the sirolimus is spray-coated on this porous surface, allowing the entire drug-loading process to be finished in less than 10 min. Recent clinical data have shown that at 9–12 months after implantation, the Yukon and Taxus stents perform similarly. A randomized optical coherence tomography study revealed that the Yukon stent significantly outperformed Cypher at a 3-month follow-up regarding neointimal thickening and strut coverage. This most likely results from the Yukon stent’s rapid sirolimus release profile and microporous surface topography. However, more thorough research is required to ascertain the Yukon stent’s long-term safety and effectiveness [85,86,87].

Despite the positive effects of DES, clinical studies have demonstrated how toxic ions produced during the breakdown of polymeric coatings or surface-coated degradable metal or metal alloys can cause inflammation. Creating a stent free of polymers would be one way to finally eliminate polymers as the drug carrier. Furthermore, carrier-free stents must be biocompatible to meld with the surrounding tissue. Polymer-free stents are anticipated to have a higher rate of drug elusion than polymeric coating-based stents, which could have a negative therapeutic impact. But, the latter ones appear to be as safe and effective as the first-generation DESs. Although polymer-free stents have been performing well in preclinical and clinical trials, these stents do not outperform second-generation DESs yet [88,89].

### 3.4. Coatings Containing Endothelial Cells

All blood vessels are lined with a single layer of endothelial cells, which control the exchanges between the bloodstream and the surrounding tissues. The growth and development of connective tissue cells, which make up the outer layers of the blood vessel wall, is orchestrated by signals from endothelial cells. Two of the most prevalent types of cardiac cells are cardiomyocytes (CMs) and endothelial cells (ECs), and they are essential for both cardiac remodeling and regeneration. Through several pathways, including the Notch and Wnt signaling pathways, ECs in the heart are crucial for the healthy development of the heart. The endothelial secretome is essential for the adult heart to maintain normal cardiac function and respond appropriately to a range of hemodynamic stimuli, such as pressure overload [90].

Given that EC damage and the exposure of the subendothelial matrix at the site of artery injury are the primary causes of thrombus and neointimal formation, it makes sense to deliver ECs on stents to diseased arterial sites for rapid re-endothelialization, EC proliferation, differentiation, and the release of growth factors, which in turn could inhibit thrombosis and neointimal hyperplasia. As a result of EC seeding, some animal studies have also demonstrated improved re-endothelialization and neointimal hyperplasia inhibition. Seeding endothelial progenitor cells (EPCs) on the stent surface was a recent innovative attempt [91]. The on-stent cell delivery of EPCs may be a novel therapeutic tool for re-endothelialization and lowering the risk of LST and ISR, according to the findings of in vitro experiments. Products like the ComboTM bioengineered sirolimus-eluting stent and the GenousTM capture R stent are made by OrbusNeich Medical, Inc. (Fort Lauderdale, FL, USA) [92]. Monoclonal antihuman CD34 antibodies are applied to the luminal surface of the 316L stainless steel R stent BMSs of the genus stent to capture EPCs. This has been tested in several clinical trials, including HEALING FIM, HEALING II, GENIUSSTEMI, and TRIAS, and it has shown promise in patients who were stable. However, because CD34+ markers used to phenotype EPCs are nonspecific, this technology may also sequester unwanted cells, including smooth muscle progenitor cells, leading to neointimal proliferation. The Combo stent has an additional abluminal coating with low-dose sirolimus, a biodegradable SynBiosys polymer, and an EPC-capture coating on the luminal surface. The REMEDEE trial is currently conducting a clinical evaluation of the Combo stent. However, the rapid loss of seeded ECs, EC damage during stent expansion, and the challenge of maintaining EC adherence to the artery wall due to blood flow have all impeded the further development of this concept [93].

A particular stent coating with a periodic structure would affect the growth and proliferation of ECs since surface topography and surface chemistry are two important elements for the adherence of proteins and cells. Compared to random nanostructured surfaces or microscale patterns, it has been shown that a larger percentage of endothelium coverage is achieved on nanoscale patterns. Consequently, adding nanopatterns to the stent surface is a viable way to speed up the re-endothelialization process. It is important to note, nonetheless, that pure nanoscale (<100 nm in both the lateral and vertical scale) surface features do not promote increased EC adhesion density as much as submicron (>100 nm in the lateral scale) titanium surface features do [94].

## 4. Metallic Coatings for Stents

### 4.1. Titanium-Based Coatings

Due to its great biocompatibility and inertness as compared to other metals, such as stainless steel, titanium is a commonly utilized material for medical devices [95]. TiOxNy-coated stents have improved mass loss, restenosis, and target vascularization outcomes while reducing platelet adhesion and fibrinogen binding. According to reports, these coatings are an excellent alternative that greatly lessens the main disadvantages of BMSs (titanium enhances biocompatibility, is inert, and has excellent corrosion resistance; titanium oxide enhances the stent’s compatibility with blood and living cells; nitrogen in this structure reduces platelet adhesion and fibrinogen binding, etc.). In addition to lowering toxicity and inflammation, the TiOxNy coating stops nickel, molybdenum, chromium, and other metals from migrating off the stainless steel surface. Thinner coatings based on TiOxNy have a mechanical issue. Although thicker films adhere to the substrate less well, they are more biocompatible. This causes coating flaws and reduces hemo- and histocompatibility. About 17.4% of the inner surface of the TITANOX stent is not covered by a protective coating. Increased stent surface protection would lower the chance of blood clots and blood artery re-narrowing [96]. Enhancing the quality of TiOxNy stent surfaces requires the further development and optimization of the current deposition process, physical vapor deposition. Coatings with varying characteristics can be achieved because of the coating’s variability of the Ox/Ny ratio. The quantity of nitrogen and oxygen in the coating affects the morphology, mechanical, and biocompatible qualities, and surface topography. As the nitrogen level rises, the stents become less thrombogenic. The low degree of dissolution in saline solution (NaCl), strong corrosion resistance, and inertness of titanium oxynitride coatings are demonstrated by their solubility study. When SBFs interact with albumin, ions, particularly calcium phosphates, are shown to be adsorbed and deposited [97].

Improved biocompatibility stents have been made from various materials, including titanium, tantalum, nickel–titanium, platinum–iridium, cobalt–chromium, and magnesium alloys. Different passivation processes to produce oxide films on the metal surface have been proposed to improve stainless steel’s performance and corrosion resistance [98].

The discrepancies observed in the published data regarding the biocompatibility of oxide and diamond-like coatings may be attributed to differences in surface modification and analysis techniques. There have been verified titanium oxide (TiO), zirconium oxide (ZrO), and diamond-like carbon (D) coatings made by plasma vapor deposition (PVD) with an unbalanced magnetron sputtering method (Teer Coatings) in response to the most recent data. This method allows for the regulation of the coatings’ chemistry, thickness, and surface shape. In vitro, platelet adhesion and fibrinogen adsorption measurements on TiO and ZrO coatings have revealed much lower values than on D coating. Compared to D-coated and uncoated St stents, both oxide-coated stents demonstrated a reduced inflammatory response and more complete endothelialization. According to the data, there may have been a notable increase in neointima surrounding D-coated stents eight weeks after stenting. This could have been caused by the D coating’s mechanical instability and poor haemocompatibility in an in vivo setting [83].

A magnesium-zinc (Mg-Zn) binary alloy was coated with a titanium dioxide (TiO_2_) nanofilm in a recent study as a possible new platform for BVS. All human tissues contain zinc (Zn), one of the most prevalent nutritionally necessary elements in the human body [99]. Zinc is a necessary element for life but has potent anti-atherogenic qualities. Moreover, Zn has been applied to enhance the mechanical qualities of magnesium for use in industry. According to reports, the analysis of viscera histology and biochemical measurements has demonstrated that the degradation products of magnesium zinc would not harm major organs [29].

Additionally, in vitro cytotoxicity tests using an L929 cell line have demonstrated the good biocompatibility of the magnesium zinc alloy. However, a significant drawback of a Mg–Zn binary alloy is its high corrosion rate, which generates H_2_ gas both in vitro and in vivo. Modifying a Mg–Zn alloy’s surface using coating technologies to reduce the initial corrosion rate is advised. To facilitate the implants’ biodegradation and full absorption by the human body, the coatings ought to function as a barrier against corrosion at various phases [100].

To control the implant device’s overall corrosion rate and prevent bodily harm during the entire process, the coatings should ideally also gradually deteriorate. Metal–metal coatings, chemical vapor deposition (CVD), ion beam-assisted deposition (IBAD), atomic layer deposition (ALD), pulsed laser deposition (PLD), etc., are examples of potential coating technologies for biomaterials. Complex shapes cannot be coated using coating technologies like IBAD and PLD because they require a line of sight for deposition. Conversely, atomic layer deposition (ALD) offers each primary surface a controlled, homogeneous, chemically bonded coating without pinholes. Internal structures can likewise be coated conformally because ALD does not depend on a line of sight. While vapor deposition (DVD) and ALD can produce chemically bonded coatings, ALD is distinguished by its capacity to divide binary reactions into two self-limiting half-reactions on the substrate surface. Furthermore, ALD reactions are highly reproducible, self-terminating, and precisely tunable in thickness through deposition cycles, making them suitable for use with delicate substrates like biomaterials [101].

In a study, ALD was selected to deposit a nanoscale thin film coating on structural Mg–Zn binary alloys that were inspired by biology. To deposit TiO_2_ on the alloys, tetrakis(dimethylamido)titanium (TDMATi) was selected as the ALD precursor. TiO_2_ nanoparticles are widely used in many everyday products, including food coloring, plastics, sunscreens, antifouling paints, and pharmaceutical additive agents. According to the FDA, TiO_2_ can be added to food products in amounts up to 1% by weight without posing any risks. TiO_2_ is also utilized in oral pharmaceutical formulations, and nano-sized TiO_2_ is regarded as a non-toxic excipient in the pharmaceutical excipients handbook (of course, depending on concentration). TiO_2_ has the potential to act as a protective barrier for the Mg–Zn substrates because it has demonstrated strong corrosion resistance on steel surfaces through the use of the sol–gel method. Before this, a 316LVM steel base for vascular stents had an ALD coating of TiO_2_, with coating temperatures adjusted to compare the mechanical characteristics of various samples [14]. Temperature increases were found to negatively impact corrosion resistance, and temperatures above 300 °C will dramatically reduce the hardness of the material. Therefore, this investigation selected 150 °C and 200 °C as the coating temperatures for the TiO_2_ deposit. TiO_2_ was chemically bonded to the substrate (Mg–Zn) by purging both TDMATi and H_2_O into the reaction chamber. Studies on the surface morphology and biocompatibility were carried out on Mg–Zn alloys coated with TiO_2_ by ALD and control samples of Mg–Zn alloys not treated by ALD [102]. The findings of this study demonstrated for the first time that endothelial cell adhesion and proliferation were enhanced at 150 °C, the ALD coating temperature at which TiO_2_ was applied to the Mg–Zn alloy stents, which are used as a BVS platform for treating blocked arteries. To shield the underlying metal from exposure to immune cells in the bloodstream, the TiO_2_ nanoscale thin film served as a protective barrier. Furthermore, the protective TiO_2_ layer may slow down the bare Mg–Zn alloy’s initial degradation rate, preventing the biomaterial from becoming inoperable before the revascularization period (5–6 months) is up. Because of its unstable surface morphology and suboptimal surface energy, which did not match that of essential proteins for mediating endothelial cell attachment, an ALD coating at 200 °C did not demonstrate such positive results with cell assays. Without additional drug elution, a well-designed, fully bioresorbable implant material should stimulate endothelial cell growth. Thus, with optimal processing temperature control, ALD thin film coating technology holds great promise for use in metallic coronary stents [103].

Metal oxide coatings, like MgO and TiO_2_, are commonly used to improve the anti-corrosion properties of magnesium alloys because of their high stability and good biocompatibility. Metal oxide coatings on magnesium alloys are currently made using a variety of technologies, such as ALD, the solvothermal method, micro-arc oxidation (MAO), and plasma electrolytic oxidation (PEO) [104].

Chemically stable metal oxide TiO_2_ is used in oral pharmaceutical preparations as nanoparticles; according to the pharmaceutical excipients manual, TiO_2_ is a non-toxic excipient (depending, of course, on concentration). Due to its exceptional qualities, including its quick endothelialization, strong blood compatibility, and anti-thrombosis effect, TiO_2_ film has been widely used to alter vascular stents. In recent work, a Ti-O film on cobalt alloy using ion beam-enhanced deposition was presented in a representative paper [105]. They confirmed that the Ti-O coating’s semi-conductor nature made it an excellent blood contact material. Furthermore, TiO_2_ can be a beneficial protective barrier for Mg-based stents because it has demonstrated strong corrosion resistance and biocompatibility on conventional metal-based stents. Using a simple solvothermal technique at 160 °C, an anatase TiO_2_ nanosheet film (thickness: 50 nm) was created on degradable Mg–Zn alloy stents. The results showed that the Mg–Zn alloy degradation rates were considerably reduced by the manufactured coating. However, the literature cited above does not thoroughly assess the potential applications for TiO_2_-coated absorbable magnesium alloy stents. According to Yang et al., a TiO_2_ film was deposited on Mg–Zn alloys using the ALD method, a cutting-edge technique that uses surface sequential reactions to achieve atomic-level film deposition and has the advantage of improving thin film adhesion and surface coverage; the TiO_2_-150 °C nanoscale film prevented corrosion on the Mg alloy and encouraged the adhesion and proliferation of endothelial cells (ECs). However, due to its unstable surface microstructure and lower-than-ideal surface energy, which did not match that of key proteins for mediating EC attachment, the TiO_2_-200 °C thin film did not perform satisfactorily in cell assays. Using a simple magnetron sputtering (MS) method at room temperature, Hou et al. successfully prepared a 400 nm thick TiO_2_ coating on a Mg–Zn alloy. They then assessed the material’s corrosion resistance and biocompatibility. After 14 days of soaking in simulated body fluid (SBF) solution, the results indicated that the degradation behavior of magnesium alloys was suppressed and that the degree of the degradation of magnesium alloys coated with TiO_2_ was not serious. Furthermore, the TiO_2_ coating promoted EC adhesion and demonstrated improved anti-platelet activity with a lower hemolysis ratio (HR, <1%). Nevertheless, the synthesized Ti-O film had a weak bond with the substrate and was extremely thin (nanoscale) [95,106].

### 4.2. Magnesium-Based Coatings

A novel technique for the in situ growth of ceramic oxide film on a valve’s metal surface (Al, Mg, Ti, and their alloys) is called micro-arc oxidation (MAO). The anti-corrosive and biocompatible qualities of magnesium-based materials can be significantly improved by implementing an MAO strategy, which can also greatly increase the MAO coating’s sustained adhesion to the substrate [29,107]. Following the preparation of MAO coatings (primarily MgO) on Mg–RE and Mg–Zn–Ca alloys, human blood was used in both static and dynamic experiments to assess the hemocompatibility of the Mg substrate and MAO coating [99]. The findings showed that in the static tests, two different types of magnesium alloys produced corrosion byproducts and showed signs of corrosion, which resulted in an incorrect evaluation of platelet adhesion. When compared to uncoated samples, all test methods (static and dynamic experiments) consistently showed that MAO coating reformed blood compatibility by reducing platelet adhesion.

To assess their biocompatibility, four bioactive MAO coatings on Mg-0.8Ca alloy using a Ca/P-based electrolyte with additional silicon (Si) and fluorine (F) were designed by Santos-Coquillat et al. [108]. For the ECs, F incorporation was essential, and the formation of a structured monolayer of ECs was inhibited by the MAO coating with a high F content (~9%–11%) and roughness (Ra ≥ 3.6 μm). Contrastingly, Lu and colleagues [109] have investigated the corrosion resistance and HR of a WE 42 alloy coated with MAO. The results of the electrochemical tests suggested that the magnesium alloy treated with MAO technology had improved corrosion resistance. However, after four weeks of immersion, the MAO coating was severely damaged. The results of a hemolysis test showed that the HRs of the WE42 and MAO groups were 50.37% ± 0.42% and 3.67% ± 0.47%, respectively, suggesting that the MAO coating is hemocompatible. Additionally, Echeverry-Rendon et al. [110] looked into how the topography and surface chemistry of pure magnesium coated with MAO affected the biological reaction of vascular cells. The outcomes showed that the MAO coating affected the vascular cells’ survival ability. SMCs and human umbilical vein endothelial cells (HUVECs) were more vulnerable to variations in Mg. The in vitro evaluation has certain limitations when predicting material properties. Therefore, more in vivo research is required to determine how vulnerable HUVECs are to the components of coated Mg.

Furthermore, the protective effect on the magnesium alloy is diminished by the typical porous microstructures of the MAO coating. A subsequent treatment is typically applied to coat the polymer coating to seal the micropores and prolong the degradation period. However, because MAO coating is a hard, rigid coating, it might not be able to provide the elastic deformation required for a magnesium alloy to be used as a vascular stent.

The loosely structured Mg(OH)_2_ is the main degradation product of materials based on magnesium. Therefore, to improve the corrosion resistance of magnesium alloy, researchers have tried to produce a homogeneous and compact hydroxide layer based on magnesium. The hydroxide films made from Mg(OH)_2_ and layered double hydroxide (LDH) have received special attention. Hydrothermal treatment is a technique in which the precursor is put in an autoclave to react at a high pressure and temperature [111].

To create a hydroxide film that improves corrosion resistance, Wang et al. synthesized a film on ZE21B alloy using the alkali-heat method (AH), which involves submerging the magnesium alloy in a 5 M boiled NaOH solution for three hours. This procedure is crucial for removing oil and other impurities from magnesium-based materials. As the results regrettably showed, the over-alkali pH value and the significant amount of Mg^2+^ released may have contributed to the ZE21B’s high cytotoxicity. In addition, the Cl^−^ ion in the solution can also break down the Mg(OH)_2_ layer, transforming it into soluble magnesium chloride (MgCl_2_). To effectively isolate the magnesium substrate from an aqueous solution or physiological environment, Mg(OH)_2_ coating is used as a physical barrier; however, this is insufficient to give magnesium alloys long-term corrosion resistance. The adhesion between the magnesium substrate and the outer layer is frequently increased, using Mg(OH)_2_ film as an inner layer [112].

### 4.3. Layered Double Hydroxide (LDH) Coatings

The interlayer structures of LDH coatings, a kind of anionic clay with a highly adjustable brucite structure, are used to resist Cl^−^ and can store anions. The general formula of LDH can be expressed as M^2+^_1−*x*_M^3+^*_x_*(OH)_2_A^n−^*_x_*/n•mH_2_O, where M^2+^ and M^3+^ are divalent and trivalent metal cations; *x* is the molar ratio of M^3+^/(M^2+^ + M^3+^) (ranging from 0.2 to 0.33), and A^n−^ represents the interlayer anions, such as NO_3_^−^, Cl^−^, PO_4_^3−^, and CO_3_^2−^. A hydrothermal treatment was successfully applied to the magnesium alloy Jiao Da Bio-magnesium-JDBM (Mg–Nd–Zn–Zr) alloy that had been coated with Mg(OH)_2_. According to Tafel curves and hydrogen evolution tests, the LDH coating greatly aided the ECs’ adhesion, migration, and proliferation in vitro and had good corrosion-resistant properties. The LDH coating’s low heart rate (<5%) satisfies all clinical application requirements. The in vivo results showed that the LDH coating containing Mg(OH)_2_ held promise for improving the anti-corrosion performance and biocompatibility because it produced the least amount of inflammation and the longest-lasting corrosion protection compared to the other samples. Through hydrothermal treatment, the research team developed an LDH film to seal the micropores of the AZ31 Mg alloy coated with the MAO layer. A two-layer structure with an inner MAO coating (~5 μm) and an outer LDH coating (~2 μm) was made possible by the MAO/LDH hybrid coating. The outcomes showed that the MAO/LDH coating prevented the magnesium alloy from degrading at a faster rate by acting as a physical barrier. The HR test revealed that the MAO/LDH coating’s HR value was 1.10% ± 0.47%, which indicates blood compatibility. However, due to its low abrasion resistance, the LDH coating’s nano-microstructure readily peeled off, and the exfoliated microstructures could enter the bloodstream and have a negative impact on the body [113].

## 5. Inorganic, Nonmetallic Coatings

### 5.1. Phosphate Coatings

Phosphate coatings, which are environmentally friendly, insoluble in water, and have high chemical stability, have recently been suggested as a viable biomedical coating substitute for chromate coatings. For the JDBM alloy, a chemical conversion Mg_3_(PO_4_)_2_ coating with a lamellar structure of roughly 1–4 μm width was designed to improve the alloy’s biological response and corrosion resistance [114]. The phosphate coating significantly reduced the rate of degradation. Moreover, an in vitro cytocompatibility test revealed that the HUVEC viability, growth, and proliferation were minimally harmed by the coated JDBM alloy. Furthermore, a decreased hemolysis ratio and the superior anti-platelet adhesion characteristic of the phosphate-coated JDBM stent were validated by an in vitro hemocompatibility test. The phosphate coating demonstrated good biocompatibility; however, its high brittleness might not be appropriate as a coating for magnesium alloy vascular stents [115].

### 5.2. Fluoride Conversion Coatings

The process of fluorination treatment is used to create a fluoride conversion film on magnesium substrates. Reaction (1) illustrates the corresponding mechanism of MgF_2_ film formation [116].

Mg^2+^ + 2F^−^ → MgF_2_↓ (1)

In the industrial and biological domains, fluoride modification has been figuratively reported to restore magnesium alloys’ resistance to corrosion. It has been used to coat the JDBM magnesium alloy with a MgF_2_ conversion layer by applying hydrofluoric acid (HF) and then the degradation and biocompatibility of the alloy was investigated in vitro [117]. Electrochemical tests revealed that the formed MgF_2_ protective layer significantly improved the Mg substrate’s degradation behavior. Additionally, the MgF_2_ coating’s HR (hemolysis ratio) was lower (10.1%), and its cytotoxicity was significantly reduced. Additionally, the MgF_2_ coating demonstrated a strong ability to inhibit platelet adhesion. However, HF treatment is not a green technique and can negatively impact lab workers’ lives and health. Mao et al. [118] created a straightforward, eco-friendly method for creating nanoscale MgF_2_ films on JDBM (Mg–Nd–Zn–Zr) alloy in 5.8 g/L of potassium fluoride solution (KF). In artificial plasma, the corrosion rate of the MgF_2_ sample was about 20% lower than that of the Mg substrate (0.269 mm/y vs. 0.337 mm/y). The researchers also verified that, when coated with the prepared MgF_2_ coating, the nanoscale structures (200–300 nm) offered a much more favorable surface that could facilitate the adhesion, proliferation, and spreading of ECs [119].

Moreover, the JDBM stent showed exceptional radial strength and compliance. The modified stent was safe and effective in vivo, as evidenced by the lack of symptoms of thrombosis or significant in-stent restenosis in the angiography pictures of the MgF_2_-coated JDBM stent. The coated stent was well-apposed to the vessel wall, and no evidence of strut fracture, in-stent restenosis, or thrombosis was detected by the follow-up intravascular ultrasonography (IVUS). Additionally, histological examination showed that the JDBM stent’s re-endothelialization into the denuded artery was facilitated by the MgF_2_ coating, confirming the biodegradable stent’s high tissue compatibility and tolerable mechanical durability. However, MgF_2_ is often used as a pretreatment method before organic coatings due to its thinness and ease of destruction [120].

### 5.3. Carbon-Based Coatings

A carbon coating, particularly diamond-like carbon coatings, is another often utilized St surface modification that is distinguished by its superior biocompatibility and chemical inertness. Additionally, it has been claimed that a covering resembling diamond lessens the release of metal ions and lowers the thrombogenicity of stainless steel. However, information on the carbon-coated stents’ performance is debatable [121].

Graphene oxide (GO) is a single-carbon nanomaterial possessing a high specific surface area, favorable mechanical characteristics, and biocompatibility. The hydroxyl (-OH), carboxyl (-COOH), and epoxy (-CH(O)CH-) groups on the GO surface, among other oxygen-containing groups, can increase the anti-corrosive ability of the magnesium alloy when GO is coated onto the Mg surface [122]. In addition, extracellular matrix (ECM) proteins can be adsorbed by GO through non-covalent interactions, hydrogen bonds, or electrostatic attraction, which encourages cell adhesion and proliferation. Additionally, to increase the biological activity of other molecules, GO’s active groups can be linked to them. Using a layer-by-layer assembly (LbL) technique, it was suggested to cover an alkali-treated magnesium alloy with a chitosan (CS)/heparinized graphene oxide (HGO) mixture. The findings of the electrochemical measurement, the change in pH, and the release of Mg^2+^ indicated that the multilayer coating significantly improved the magnesium alloy’s resistance to corrosion [123].

CS/HGO multilayer coating has the potential to significantly lower platelet adherence while promoting EC adhesion and proliferation. The LbL method is a novel and simple surface treatment technology that uses electrostatic interactions between opposite charges to expose the charged substrate to positively and negatively charged polyelectrolyte solutions in alternate ways [124]. Furthermore, LbL films on the magnesium alloy can achieve multi-functionalized coating (drug release, self-healing characteristics, etc.) with varying polyelectrolytes and cycle times. As we will cover in more detail below, the LbL method is currently more frequently employed in creating organic or polymer coatings on Mg-based materials [125].

As a 1D graphite structure, GO possesses a sizable specific surface area [126]. Due to its abundance of functional groups, GO may be modified in various ways and has a high capacity for the covalent and physical adsorption of medicines with a negative charge. Consequently, GO has found extensive application in the field of biomedical engineering as a medication carrier. GO has been made using a modified Hummer method that includes the active anticancer medication chlorogenic acid (CA). About 13.1% of the CA in the nano-mixture was loaded. When the reduced GO was mixed with polyethylene glycol (PEG), it was discovered that PEG–BPEI–rGO was able to load more doxorubicin through hydrophobic contact and the π–π bond than the unreduced GO and PEG. In addition to acting as a drug carrier, GO has worked in the fields of cardiology and other sciences. Jing et al. used electrospinning to create a small-caliber artificial blood artery made of thermoplastic polyurethane–GO that may encourage cell adherence and development on the surface. These findings can suggest a different course for the use of GO in the management of cardiovascular conditions. However, there are still a few uses of GO in this field. This investigation has shown that the GO double-layer drug-coated stents are resilient and do not experience shedding [127]. The stents with different coatings may effectively stop the growth of VSMCs and have good blood compatibility. The adherence and activation of platelets and the migration and proliferation of VSMCs are markedly inhibited by GO double-layer drug coating [128]. The in vivo rabbit investigation has shown that GO coating encourages endothelialization and suppresses thrombosis and intimal hyperplasia. The results of the toxicity tests indicate that the coating has no discernible impact on the primary physiological markers of zebrafish embryos, such as survival rate, hatching rate, and heart rate. Zebrafish embryos and young fish do not develop abnormally due to GO double-layer drug-coated stents. To achieve anti-proliferation and anti-thrombosis, there have been stents created that are coated with a novel type of GO double layer [129].

The modification of 316L stainless steel (316L SS) stents with dopamine (DA) involves coating the outer layer with carboxymethyl chitosan (CMC) loaded with heparin (Hep) and the inner layer with GO loaded with docetaxel (DTX). Numerous in vitro and in vivo tests have been conducted to confirm its efficacy and biosafety. A new coating for 316 alloy stainless steel stents was created using an easy-to-use and eco-friendly process. A new coating layer was created on the stent with a thickness of 6 and 10.6 μm, made of graphene sheets that were immediately exfoliated in chitosan and then adorned with TiO_2_NPs. Compared to the uncoated and chitosan-coated stents, the coated stent based on graphene sheets has encouraging favorable mechanical and hematological properties. Additionally, a study was conducted on the mechanical properties of the created coating layer. When exposed to healthy and diabetic human blood with excellent stability, the stent coated with graphene sheets demonstrated no blood platelet attachment. The created stent did not have any deleterious effects on the count of red blood cells or white blood cells. This work will pave the way for developing green synthetic graphene sheets for stent coating, extending the device’s useful life [130].

## 6. Summative Discussion and Future Perspectives

One of the leading causes of death in the modern world is CVD, taking an annual toll that has been rising without additional improvement in recent years. The vascular stent has been one of the most successful medical treatments for peripheral and coronary artery problems, advancing over the years from BMSs to several DES generations and eventually leading to the emergence of BVSs.

Using the antiproliferative drug as a coating on a balloon’s surface as an alternative to a standard stent platform is an interesting solution, where prolonged 60 s inflation is used to locally provide medication to the tissue. This method is particularly appealing for treating de novo lesions in small-caliber vessels [131,132,133,134,135]. Moreover, drug-coated balloons can be used when adding an additional stent layer is not desired, like in bifurcation lesions or cases involving multiple stent layers [136]. However, while the results of this procedure for curing restenosis are comparable to those of implanting a second layer of DESs, their use is limited because they have a significantly greater cost [131]. The currently available evidence indicates a significant degree of comparability between the use of drug-coated balloons and DESs in patients experiencing restenosis with BMSs in terms of both clinical efficacy and safety profiles [137]. On the one hand, the method of choice in these situations is the use of drug-coated balloons, mainly because they do not have a second strut layer. On the other hand, in the more complicated case of intrastent restenosis in patients who have received DES, the use of DES for therapy shows a marginally higher efficacy than the use of drug-coated balloons, especially in terms of the need for target lesion revascularization [136,138,139].

**Figure 3:**
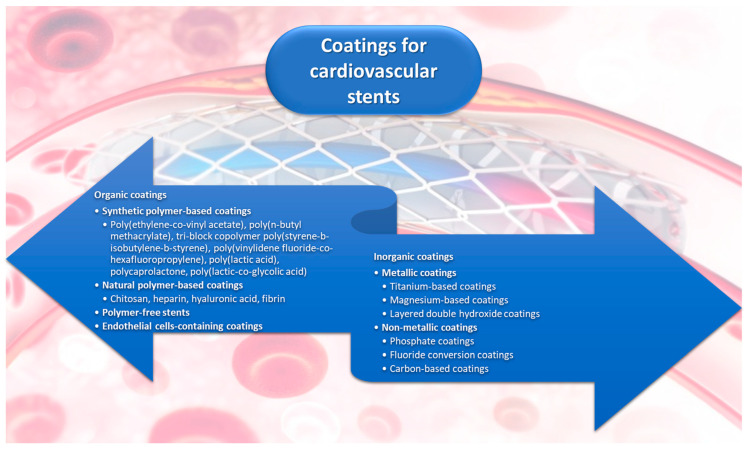
Summary of coating possibilities for cardiovascular stents.

To minimize the harmful potential of DES and ensure optimum therapeutic efficacy, much attention must be given to the coating material. Covering the metal structure of the stent with an appropriate coating allows for the controlled release kinetics of the incorporated drug, uniform dosage, and structural integrity for the device. To fulfill these roles, the material utilized to coat these biomedical devices must be biocompatible, non-thrombogenic, non-inflammatory, and non-toxic to cells to promote arterial healing and reendothelialization, being the interface between the stent and the vascular tissue. To meet all these important criteria, worldwide research has been put into elaborating various stent coating compositions based on several organic and inorganic components, as summarized in Figure 3 and Table 1.

Despite the existence of numerous coating alternatives in different testing stages, research continues to optimize DES design and subsequential performance [143]. In addition to advancing some of the recently reported above-mentioned coating formulations to later evaluation stages, several other future perspectives have been envisaged lately. For instance, one recommended therapeutic strategy consists of utilizing drug-filled stents (DFSs) that can ensure controlled elution from an internal stent lumen without requiring a polymer layer. DFS early trial results have revealed a high degree of stent strut coverage, little neointimal hyperplasia, and good clinical outcomes at one month after implantation [131]. Another important problem that is soon to be solved is the creation of personalized stents [40]. In this respect, much work has been directed toward implementing 3D printing techniques for creating customized structures with controlled topography and optimum coating adhesive surfaces that would perfectly fit the specific needs of each patient [135,148]. Additionally, there is an increasing interest in developing stents coated with materials that can also be visualized on X-rays. It has been suggested that the incorporation of common X-ray contrast agents (e.g., barium salt, iodine compound, and zirconia) may be an efficient method for increasing the radiopacity of polymeric structures. This is a desirable feature for coronary stents (especially for non-metallic platforms). These cutting-edge imaging methods could open up new avenues for demonstrating the stent’s extension, location, and degradation degree, enabling more thorough stent monitoring than marker immobilization [40].

## 7. Conclusions

To summarize, a plethora of coatings have been described, ranging from synthetic and natural polymers to metallic and nonmetallic inorganic alternatives, each with different degrees of success. PLA-based polymers are a potential class of materials for developing fully resorbable stents due to their great biocompatibility and technological appropriateness. As a result, the polymer can temporarily work mechanically before degrading. Conversion coatings (such as MAO, LDH, and MgF_2_) and deposited coatings (such as TiO_2_ and GO) are inorganic coatings with potential in cardiovascular stent optimization. Conversion coatings are typically utilized as the inner layer because of their in situ development mechanism, which forms a strong link with magnesium substrates. Deposited inorganic coatings are categorized as ex situ because they typically exhibit a weak interfacial adherence to magnesium substrates and are created by physical adsorption. They may serve as an external coating to give magnesium alloy stents certain functions. Furthermore, an effective way to encourage quick endothelialization and inhibit blood vessel hyperplasia in magnesium-based stents may be to design and produce targeted biomolecules with good biocompatibility based on the bionics of blood vessels and their microenvironments.

To conclude, being aware of emerging stent optimization possibilities represents a first step in developing more performant medical devices for better treatment outcomes. Thus, updating the literature on the advancements in organic and inorganic coatings, this review aims to serve as an inception point for future research toward creating enhanced solutions for CVD patients worldwide.

## Figures and Tables

**Table 1:** Overview of coating types, materials, and associated observations.

Coating Type	Material	Observations	References
Synthetic polymer	Poly(ethylene-co-vinyl acetate)	Used in 1st generation of DESs Loaded with sirolimus Associated with late stent thrombosis Delayed wound healing due to poor reendothelialization and persistence of polymer coatings after drug release	[39,140]
Synthetic polymer	Poly(n-butyl methacrylate)	Used in 1st generation of DESs Loaded with sirolimus Maintains chemical integrity after multiple years of stent implantation Associated with late stent thrombosis Delayed wound healing due to poor reendothelialization and persistence of polymer coatings after drug release	[39,140,141]
Synthetic polymer	Tri-block copolymer poly(styrene-b-isobutylene-b-styrene)	Used in 1st generation of DESs Loaded with paclitaxel Allows for early burst release of the drug Delayed wound healing due to poor reendothelialization and persistence of polymer coatings after drug release	[39,140]
Synthetic polymer	Phosphorylcholine	Used in 2nd generation of DESs Ability to load and release a variety of therapeutic agents Reduces platelet adhesion and subsequential thrombosis	[140,142]
Synthetic polymer	Copolymer poly(vinylidene fluoride-co-hexafluoropropylene)	Used in 2nd generation of DESs Loaded with everolimus Maintains chemical integrity after multiple years of stent implantation	[140,141]
Synthetic polymer	Poly-lactic acid	Used in 3rd generation of DESs Degrades into harmless compounds that are further metabolized by the body Reduced risk of cardiac events compared to durable polymer coatings	[39,143]
Synthetic polymer	Poly(lactic-co-glycolic acid)	Used in 3rd generation of DESs Degrades into harmless compounds that are further metabolized by the body Capable of a sustained and directional release of high-molecular biological compounds	[48,143]
Natural polymer	Chitosan	Slows corrosion rate of stent platform Can be combined with other materials (e.g., poly-L-glutamic acid, graphene oxide, heparin) to provide synergistic outcomes	[62,144]
Natural polymer	Heparin	Anticoagulant properties Inhibitory effect on arterial smooth muscle cell proliferation Reduces stent thrombosis and restenosis	[68,92]
Natural polymer	Hyaluronic acid	Significantly reduces the formation of platelet thrombus Favorable antiproliferative effect and decreased anti-inflammatory response	[73,74]
Natural polymer	Fibrin	Provides complete endoluminal paving with anti-thrombogenic or antiproliferative therapy delivery Stimulates cell adhesion, spreading, migration, and alignment	[77,81]
Metallic	TiOxNy	Improved chemical/corrosion stability Improved mass loss, restenosis, and target vascularization Reduced platelet adhesion and fibrinogen binding	[14,145]
Metallic	Titanium dioxide	Strong corrosion resistance Slows down degradation of Mg–Zn alloy-based stents Encourages the adhesion and proliferation of endothelial cells Has anti-thrombotic properties	[102,105]
Metallic	Magnesium hydroxide	Improved corrosion resistance Augmented reendothelialization Anti-inflammatory and anti-thrombotic effects	[146,147]
Inorganic, non-metallic	Graphene oxide	Reduced adherence and activation of platelets Reduced migration and proliferation of VSMCs Promotes reendothelialization Suppresses thrombosis and intimal hyperplasia	[127,128]

## Data Availability

Not applicable.
